# Etiological Factors Associated With Abnormal Uterine Bleeding Among Adult Women Presenting to Tertiary Healthcare Settings

**DOI:** 10.7759/cureus.88019

**Published:** 2025-07-15

**Authors:** Reeta Bai, Aneela Ashraf, Sadia Shoeb, Zeeshan Hussain, Maira, Farah Tabassum

**Affiliations:** 1 Obstetrics and Gynecology, Sheikh Saeed Memorial Campus, The Indus Hospital and Health Network, Karachi, PAK; 2 Underwater and Hyperbaric Medicine, Pakistan Navy Station (PNS) Shifa Hospital, Karachi, PAK; 3 Hyperbaric and Diving Medicine, Armed Forces Hospital, King Abdulaziz Air Base, Dhahran, SAU

**Keywords:** adenomyosis, endometrial polyps, etiology, leiomyoma, uterine hemorrhage, uterine hemorrhage/diagnosis

## Abstract

Background: Abnormal uterine bleeding (AUB) is a common gynecological condition affecting women of reproductive age and is associated with quality of life, physical well-being, and healthcare impact. AUB has varying etiologies classified as structural or non-structural, which influence clinical presentation and patient outcomes. The purpose of this study was to determine the prevalence of AUB etiological determinants in women of reproductive age and to evaluate the role of these sociodemographic factors affecting their prevalence.

Methods: It is a cross-sectional observational study conducted over six months in a tertiary care hospital in Karachi. Non-probability consecutive sampling was used to recruit 171 women diagnosed with AUB and aged between 18 and 45 years. Clinical examination and transvaginal ultrasound were used to assess participants. Information on ages, body mass index (BMI), parity, educational level, socioeconomic status, and place of residence was collected.

Results: The average age was 39.17 ± 5.24 years, and the BMI was 27.94 ± 5.96 kg/m^2^. Leiomyoma was the most frequent etiological factor in 171 women (56.7%), followed by polyps (28.1%) and adenomyosis (10.5%). Infrequent cases were endometrial abnormalities (1.8%), iatrogenic (2.3%), and hyperplasia (0.6%). The structural causes were more prevalent among less-educated women and among women who lived in under-resourced regions. There were no definite associations with age, BMI, marital status, or parity. The mean duration of symptoms was 15.15 ± 11.68 months, which demonstrated the tendency of late clinical manifestation.

Conclusion: Leiomyomas and polyps were found to be the most common underlying causes of AUB among reproductive-age women, where structural factors were more prevalent among less well-educated women and patients with limited healthcare access.

## Introduction

Abnormal uterine bleeding (AUB) is a common gynecological condition that significantly affects the quality of life of women at their reproductive age [1]. It is identified by alterations in volume, regularity, and timing of menstrual periods and affects 20-35% of women worldwide [2]. In low-income and middle-income countries, it is assumed to be more prevalent due to the unavailability of diagnostic facilities and specialized treatment [3]. To standardize the diagnosis of AUB, the PALM-COEIN classification was created by the International Federation of Gynecology and Obstetrics (FIGO), which categorized causes in structural (polyp, adenomyosis, leiomyoma, malignancy/hyperplasia) and non-structural (coagulopathy, ovulatory dysfunction, endometrial, iatrogenic, and not otherwise classified) groups [4]. Structural causes, especially leiomyomas and polyps, are commonly found in clinical practice and often require a more invasive approach [5].

Some demographic and clinical factors, including age, body mass index (BMI), parity, education, and residence location, have the potential to affect the presentation and diagnosis of AUB [6]. However, despite its frequent use, there is a lack of recent, domestically produced data from low- and middle-income countries on this classification structure. Consequently, it leads to the practice of making clinical decisions without etiological knowledge, resulting in healthcare burden and delays in effective healthcare treatments [7]. Therefore, identification of the most prevalent causes of AUB with the help of the PALM-COEIN system may facilitate more precise diagnosis and better treatment options [8].

The objective of this study is to assess the frequency and pattern of etiological factors of AUB among women of reproductive age. This study also aims to evaluate the relationship of these factors with clinical and demographic characteristics that may guide more formal diagnostic procedures and contribute to early, evidence-based treatment.

## Materials and methods

This cross-sectional observational study was conducted over six months (October 2023 to March 2024) at the Obstetrics & Gynecology department of the outpatient facility at the Indus Hospital in Karachi, Pakistan. The study was approved by the institutional review board (IRB) under ref 10-23/37MS. It was conducted to identify the frequency and distribution of etiological factors in AUB among women of reproductive age and to investigate the role of the sociodemographic determinants in shaping the prevalence of these etiologies. After explaining the purpose and procedures of the study, informed written consent was obtained from all participants.

A non-probability consecutive sampling approach was employed to recruit the participants. The sample size was determined using OpenEpi version 3.0.0 (release 2013, Atlanta, GA, USA). Based on previously reported frequencies of etiological causes of AUB, a total of 171 women were recruited. Inclusion criteria included women aged between 18 and 45 years who had AUB for over six months. Women with a history of ectopic or molar pregnancy, current or prior gynecologic malignancy, recent hormonal therapy, or anticoagulation were excluded, as these factors may individually affect bleeding patterns and confound interpretation of diagnostic testing.

Information was collected using a structured and pre-designed proforma and recorded electronically. Some of the more common variables assessed were age, BMI, parity, marital and educational status, place of residence, and socioeconomic background. PALM-COEIN standards confirmed the diagnosis of etiological factors. Each participant was evaluated based on potential etiological factors according to the PALM-COEIN classification system, which comprises both structural (PALM) and non-structural (COEIN) causes. Etiological factors recorded included leiomyoma, polyp, adenomyosis, hyperplasia, endometrial abnormalities, and etiological causes. Clinical information also included the symptoms of abnormal bleeding. All patients were evaluated by a comprehensive gynecological examination, comprising an internal pelvic examination, transvaginal ultrasound (TVUS), and endometrial biopsy. TVUS was primarily used to identify structural abnormalities, including leiomyomas, polyps, and characteristics indicating adenomyosis.

IBM SPSS Statistics for Windows, Version 26 (Released 2019; IBM Corp., Armonk, New York), was used to analyze data. The mean, standard deviation, frequency, and percentages were computed using descriptive statistics to summarize demographic and clinical data. Categorical parameters in etiological factors and demographic variables were analyzed using the chi-square test. The p-value < 0.05 was considered significant.

## Results

The study included 171 women aged 18 to 45 years with AUB. The average age and BMI were 39.17 ± 5.24 years and 27.94 ± 5.96 kg/m², suggesting an overweight population. Etiologic leiomyoma (56.7%) was most common, followed by polyps (28.1%) and adenomyosis (10.5%). Hyperplasia, endometrial, and iatrogenic factors were less frequent. Structural (PALM) causes were more common than non-structural (COEIN). Stratification revealed no statistically significant association between etiological factors. The demographic and clinical variables are listed in Table 1.

**Table 1:** Descriptive Statistics of Demographic and Clinical Variables (n=171) SD = standard deviation, % = percentage, BMI = body mass index, kg = kilogram, cm = centimeter, CI = confidence interval

Variable	Mean ± SD	Median	Min–Max	95% CI for Mean	Skewness	Kurtosis
Age (years)	39.17 ± 5.24	40.00	20–45	38.38–39.96	-0.942	0.414
Weight (kg)	65.97 ± 14.20	66.00	24–98	63.83–68.11	0.018	-0.329
Height (cm)	153.46 ± 6.27	153.00	130–171	152.52–154.41	0.021	0.782
BMI (kg/m²)	27.94 ± 5.96	27.70	16.1–42.2	27.04–28.84	0.208	-0.541
Parity (n)	2.95 ± 2.15	3.00	0–9	2.62–3.27	0.403	-0.309
Duration of disease (months)	15.15 ± 11.68	12.00	6–84	13.38–16.91	3.796	18.681

Women with AUB had a mean age of 39.17 years and a BMI of 27.94 kg/m², demonstrating an overweight population. Average weight and height were 65.97 kg and 153.46 cm. The mean parity was 2.95, and symptoms lasted for 15.15 months, suggesting that AUB is prevalent among overweight women during the late ages of reproduction. Table 2 illustrates the frequency of etiological factors associated with AUB.

**Table 2:** Frequency Distribution of Sociodemographic Characteristics (n=171) *= Significant at p < 0.05

Variable	Categories	Frequency	Percentage (%)	Test Used	Test Value	Significance
Marital Status	Married	155	90.6%	Chi-square	χ² = 2.89	p = 0.236
	Single	11	6.4%			
	Widow	5	2.9%			
Socioeconomic Status	Lower	63	36.8%	Chi-square	χ² = 4.52	p = 0.104
	Middle	93	54.4%			
	Upper	15	8.8%			
Educational Status	Illiterate	13	7.6%	Chi-square	χ² = 12.98	p = 0.024*
	Primary	74	43.3%			
	Middle	63	36.8%			
	Matric	11	6.4%			
	Intermediate	5	2.9%			
	Graduate	5	2.9%			
Residential Status	Korangi	94	55.0%	Chi-square	χ² = 15.31	p = 0.018*
	South	27	15.8%			
	West	18	10.5%			
	Malir	13	7.6%			
	East	12	7.0%			
	Others/Central	7	4.1%			
Smoking Status	Smoker	0	0.0%	-	-	-
	Non-Smoker	171	100.0%			

Most of the participants were married (90.6%). Regarding socioeconomic status, 54.4% were in the middle-income category, and 36.8% were in the lower-income category. A total of 43.3% of the respondents had primary education, and 36.8% completed secondary education. Most women (55%) lived in Korangi, while 15.8% lived in the South district. None of the participants were smokers. The analysis revealed a significant relationship between etiological subtypes and education (p = 0.024) and residence (p = 0.018). Table 3 shows the distribution of causes of AUB according to the PALM-COEIN classification.

**Table 3:** Frequency of Etiological Factors of Abnormal Uterine Bleeding (AUB) (n=171) *= Significant at p < 0.05 AUB = abnormal uterine bleeding, L = leiomyoma, P = polyp, A = adenomyosis, E = endometrial, M = malignancy and hyperplasia, N = not otherwise classified

Etiological Category (PALM-COEIN)	Frequency	Percentage (%)	Test Used	Test Value	Significance
Leiomyoma (AUB-L)	97	56.7%	Chi-square	χ² = 11.27	p = 0.024*
Polyp (AUB-P)	48	28.1%	Chi-square	χ² = 9.63	p = 0.047*
Adenomyosis (AUB-A)	18	10.5%	Chi-square	χ² = 3.10	p = 0.540
Endometrial (AUB-E)	3	1.8%	Chi-square	χ² = 1.52	p = 0.823
Iatrogenic/Not Otherwise Classified (AUB-N)	4	2.3%	Chi-square	χ² = 2.34	p = 0.674
Hyperplasia (AUB-M)	1	0.6%	Chi-square	χ² = 0.89	p = 0.927

The most common cause was leiomyoma (56.7%), followed by polyp (28.1%) and adenomyosis (10.5%). The least frequent causes were iatrogenic (2.3), endometrial (1.8), and hyperplasia (0.6). More common were structural causes indicating the statistically significant relationships between the etiological categories and education (p = 0.024) and residential status (p = 0.018). This distribution highlights structural causes as dominant in AUB etiology.

## Discussion

This study aimed to establish the etiological pattern of AUB among women of reproductive age and to identify the effects of sociodemographic factors on the etiology. The results demonstrated that structural abnormalities were most common, specifically leiomyomas and endometrial polyps, whereas non-structural adenomyosis, endometrial pathologies, and iatrogenic factors displayed reduced frequency. Moreover, education level and residence were found to be associated with AUB patterns.

Women with lower education from underserved regions were more often diagnosed with structural causes of AUB. These findings align with previous literature, specifying that a low level of education is associated with poor health knowledge and subsequent late healthcare-seeking [9]. Another multicenter study also identified that rural populations experience diagnostic delays because imaging services are less available in rural areas [10,11]. The current study observed that the most prevalent structural reason was leiomyomas, consistent with investigative studies that showed the fibroids cause over half of all AUB events in comparable demographic groups [12]. Polyps were identified as the second most prevalent cause, which is also consistent with prior reports, noting that endometrial polyps are underreported due to the lack of hysteroscopy use [13]. Because ultrasound is less sensitive than MRI, adenomyosis was underreported, even though this study discovered it in many cases. It aligns with recent data that showed the diagnostic value of MRI in the detection of diffuse adenomyosis, particularly in younger women, was higher [14,15].

The study found that sociodemographic factors may support early diagnosis and management of AUB. Consistently, a study found that inadequate counseling in AUB cases may significantly affect treatment satisfaction and continuation [16]. Conversely, studies investigating community-based health education have indicated improved early diagnosis and treatment adherence [17]. Although our findings were in line with the structural-dominant pattern reported in previous studies, certain discrepancies, including the low adenomyosis level, may reflect diagnostic constraints. Moreover, the protective effect of parity on fibroids reported in prior investigations was not found in this study [18].

The limitations of this study include the single-center research design and dependency on ultrasound imaging. Due to the cross-sectional nature of the study, causal interpretations were not assessed. Furthermore, several possible confounding factors, including a history of using contraceptives, hormonal treatment, nutrition, and psychological stress, were not evaluated and may affect clinical manifestation and diagnosis of AUB. Future research needs to consider a prospective, multicenter methodology with standardized imaging (including MRI) and assessment of an intervention strategy that could correct diagnostic delays in underserved populations.

## Conclusions

This study demonstrated that the most prevalent etiological causes of AUB among women of reproductive age were structural reasons, especially leiomyomas and endometrial polyps. These causes were particularly related to specific sociodemographic attributes, including a lower level of formal education and living in under-resourced or underserved areas.

The non-structural factors, including adenomyosis, iatrogenic influences, and endometrial irregularities, were relatively less prevalent outcomes, probably due to the known constraints of the diagnosis and limited sensitivity of standard imaging techniques like ultrasound.
